# Determination of acrolein generation pathways from linoleic acid and linolenic acid: increment by photo irradiation

**DOI:** 10.1038/s41538-022-00138-2

**Published:** 2022-04-12

**Authors:** Shunji Kato, Naoki Shimizu, Yurika Otoki, Junya Ito, Masayoshi Sakaino, Takashi Sano, Shigeo Takeuchi, Jun Imagi, Kiyotaka Nakagawa

**Affiliations:** 1 J-Oil Mills Innovation Laboratory, Graduate School of Agricultural Science, Tohoku University, Sendai, Miyagi 980–8572 Japan; 2 Food and Biodynamic Chemistry Laboratory, Graduate School of Agricultural Science, Tohoku University, Sendai, Miyagi 980–8572 Japan; 3 Food Design Center, J-OIL MILLS, INC., Yokohama, Kanagawa 230-0053 Japan

**Keywords:** Lipid peroxides, Risk factors

## Abstract

2-Propenal (acrolein) is a toxic aldehyde generated from the thermal degradation of edible oils. While previous studies have suggested that linolenic acid (LnA) is the origin of acrolein formation in edible oils, these studies were performed under thermal conditions where only the fatty acid hydroperoxide (FAOOH) isomers derived from radical oxidation were formed. In this study, we reinvestigated the acrolein generation pathway through another oxidation mechanism involving singlet oxygen (^1^O_2_) oxidation (type II photo-oxidation). Standards of the main FAOOH isomers (oleic acid hydroperoxide, linoleic acid hydroperoxide (HpODE), and linolenic acid hydroperoxide (HpOTE)) found in edible oils were prepared, and their decomposition products, including those derived from^1^O_2_ oxidation (i.e., 10- and 12-HpODE) were analyzed by GC-EI-MS. We found that ^1^O_2_ oxidation products of linoleic acid (LA) and LnA but not OA, are significant sources of acrolein formation. The amount of acrolein formed from edible oils high in LA (e.g., rice bran oil) increased by photo irradiation. Further investigation into the mechanism of acrolein generation demonstrated that the amount of acrolein derived from ^1^O_2_ oxidation-specific HpOTE isomers (i.e., 10- and 15-HpOTE) was two times greater than that of other HpOTE isomers (i.e., 9-, 12-, 13-, and 16-HpOTE). The results of the present study provide a new pathway of acrolein formation from type II photo-oxidation. This information can be used to inform on oil storage and processing conditions to reduce exposure and dietary intake of acrolein.

## Introduction

2-Propenal (acrolein, CAS No. 107-02-8) is a toxic aldehyde listed by the International Agency for Research on Cancer^1^ and the Environmental Protection Agency^2^ as an air pollutant. Due to its strong electrophilic character, acrolein potentially reacts with proteins^3^ and DNA^4^. Evidence suggests that such modification of biomolecules by acrolein contributes to the onset and development of Alzheimer’s disease^5^, cardiovascular disease^6^, and cancer^7^. The LD_50_ of acrolein is calculated to be 7–46 mg/kg body weight (rats, mice, and hamsters)^4^. Accordingly, reducing the exposure and dietary intake of acrolein is desirable for chronic disease risk reduction. Acrolein is formed during the combustion of petroleum fuels and tobacco^6^, but the thermal degradation of edible oils is among the most common sources of acrolein exposure^8–14^.

**Fig. 1:**
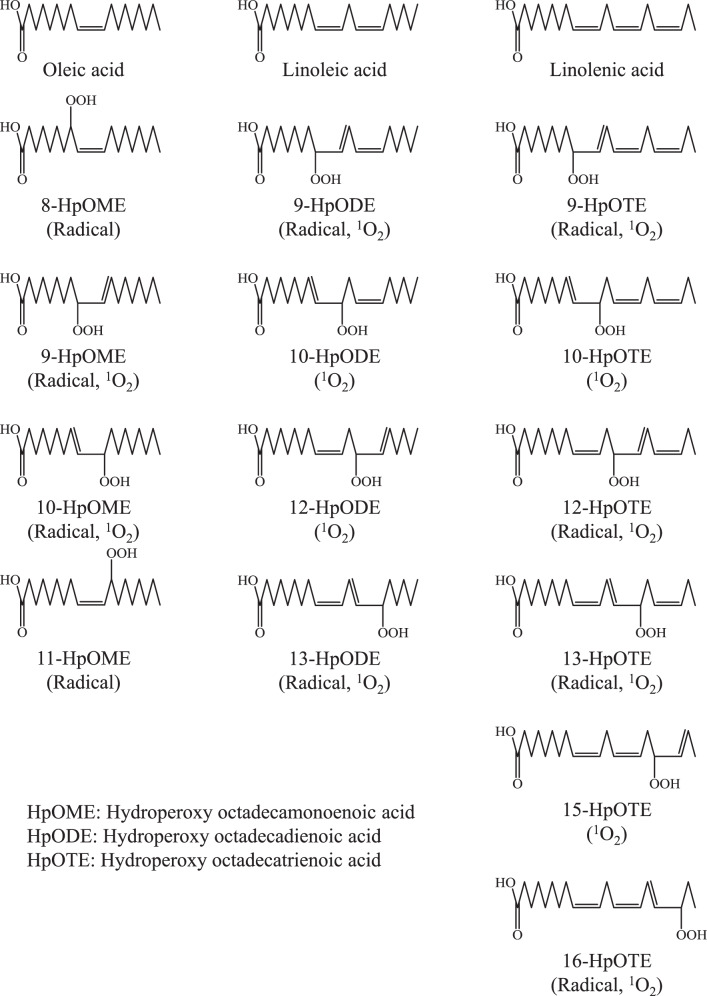
Chemical structures of OA, LA, LnA, and their hydroperoxyl group positional isomers. Each isomer is generated by radical and/or ^1^O_2_ oxidation.

Studies have shown that the decomposition of triacylglycerol (TG), the main constituent of edible oils, is a key reaction in the formation of acrolein. For example, earlier studies revealed a pathway where TG is first hydrolyzed to glycerol and subsequently dehydrated to form acrolein^15,16^. However, in an isotope labeling study, Alice et al., demonstrated that the glycerol backbone of TG is not the predominant source of acrolein^17^. Subsequently, more recent studies have suggested that the oxidation of the fatty acid (FA) moiety, especially oxidation of α-linolenic acid (LnA) rather than oleic acid (OA) and linoleic acid (LA), contributes to the formation of acrolein^10^. For example, one study showed that during heating, the amount of acrolein derived from LnA was ten times higher than that derived from LA, and that only a negligible amount of acrolein was formed from OA^17^. Such formation of acrolein from the FA moiety of TG has been suggested to initiate via the radical oxidation of FA, which affords FA hydroperoxide (FAOOH) positional isomers (Fig. 1). In the case of LnA, four hydroperoxy octadecatrienoic acid (HpOTE) isomers (9-HpOTE, 12-HpOTE, 13-HpOTE, and 16-HpOTE) that possess hydroperoxyl groups at different positions are produced by radical oxidation. Following this radical oxidation, acrolein is formed through a series of decomposition reactions (e.g., β-scission) that initially occurs at the hydroperoxyl group of FAOOH isomers. Hence, on the basis of this pathway, FA molecular species and the hydroperoxyl group position of FAOOH can be considered as a key factor during the formation of acrolein in edible oils.

Previous studies on the formation of acrolein from edible oils have mainly focused on pathways that initiate from radical oxidation. Although it has been traditionally considered that radical oxidation is the main mechanism involved in edible oil oxidation, recent studies have demonstrated the significant contribution of another oxidation mechanism, ^1^O_2_ oxidation, towards the oxidation of edible oils. For example, we recently demonstrated that commercial edible oils are oxidized by Type II photo oxidation (i.e., ^1^O_2_ oxidation) and contains a significant amount of ^1^O_2_ oxidation-specific FAOOH isomers (i.e., hydroperoxy octadecadienoic acid; HpODE, the oxidation product of LA), even when analyzed immediately after opening^18^. Importantly, radical oxidation and ^1^O_2_ oxidation each afford FAOOH isomers that differ in their positions of the hydroperoxyl group (Fig. 1). Hence, assuming that FA molecular species and the hydroperoxyl group position of FAOOH affect the formation of acrolein, a novel acrolein formation pathway that initiates from the ^1^O_2_ oxidation of LnA and/or other FA molecular species can be suggested.

In this study, the decomposition of the hydroperoxides of the main FA in edible oils (i.e., OA, LA, and LnA) from thermal or photo-oxidation was investigated. FAOOH isomer standards were prepared by both ^1^O_2_ and radical oxidation (Fig. 1) and were thermally decomposed. Acrolein and other volatile compounds generated from each FAOOH isomer were analyzed using gas chromatography mass spectrometry (GC-MS) to reinvestigate the acrolein generation pathway. We found that oxidation products of OA did not generate acrolein, whereas LnA and LA generated acrolein via ^1^O_2_ oxidation. This was further confirmed via the analysis of photooxidized edible oils. The results of this study provide insights into the mechanisms of oil oxidation and have practical implications for food storage and processing.

## Results and discussion

### Preparation of FAOOH hydroperoxyl group positional isomers

OA, LA, and LnA, the main unsaturated FA contained in edible oils, are oxidized during the oxidation of edible oils. Oxidation of FA affords FAOOH positional isomers based on the oxidation mechanism involved (i.e., radical oxidation and ^1^O_2_ oxidation, Fig. 1). Considering that acrolein is formed through a series of decomposition reactions (e.g., β-scission) that initially occurs at the hydroperoxyl group of FAOOH isomers, FA species and oxidation mechanisms may determine the formation pathway of acrolein. However, due to the lack of reliable standards of each FAOOH isomer, FAOOH isomers and pathways that contribute to the formation of acrolein has not been thoroughly investigated. Therefore, it was critical to prepare high purity FAOOH isomer standards. The isomeric profile of FAOOH formed by each oxidation mechanism is summarized in several studies^19^. According to such studies, the possible HpOME isomers were all generated by radical oxidation, whereas the possible HpODE and HpOTE isomers were all generated by ^1^O_2_ oxidation. Therefore, to prepare each FAOOH isomer standard, OA was oxidized by radical oxidation using azobis (4-methoxy-2,4-dimethylvaleronitrile) (MeO-AMVN) as a radical initiator, while LA and LnA were oxidized by ^1^O_2_ oxidation using rose bengal as a photosensitizer.

When OA was oxidized by a radical, OA generated several secondary oxidation products together with HpOME because the energy required to abstract an allylic hydrogen is high enough to decompose a hydroperoxyl group^20^. Therefore, to remove secondary oxidation products, a crude radical oxidation product of OA was purified with reverse phase HPLC (Supplementary Fig. 1A). The purified HpOME fraction was further separated to each HpOME isomer by normal phase HPLC (Supplementary Fig. 1B). On the other hand, because ^1^O_2_ selectively reacts with a double bond via an ene-reaction at low reaction temperatures, the decomposition of hydroperoxides can be avoided. Accordingly, oxidation products of LA and LnA were directly purified by normal phase HPLC to HpODE and HpOTE isomers, respectively, (Supplementary Fig. 1C, D).

**Fig. 2:**
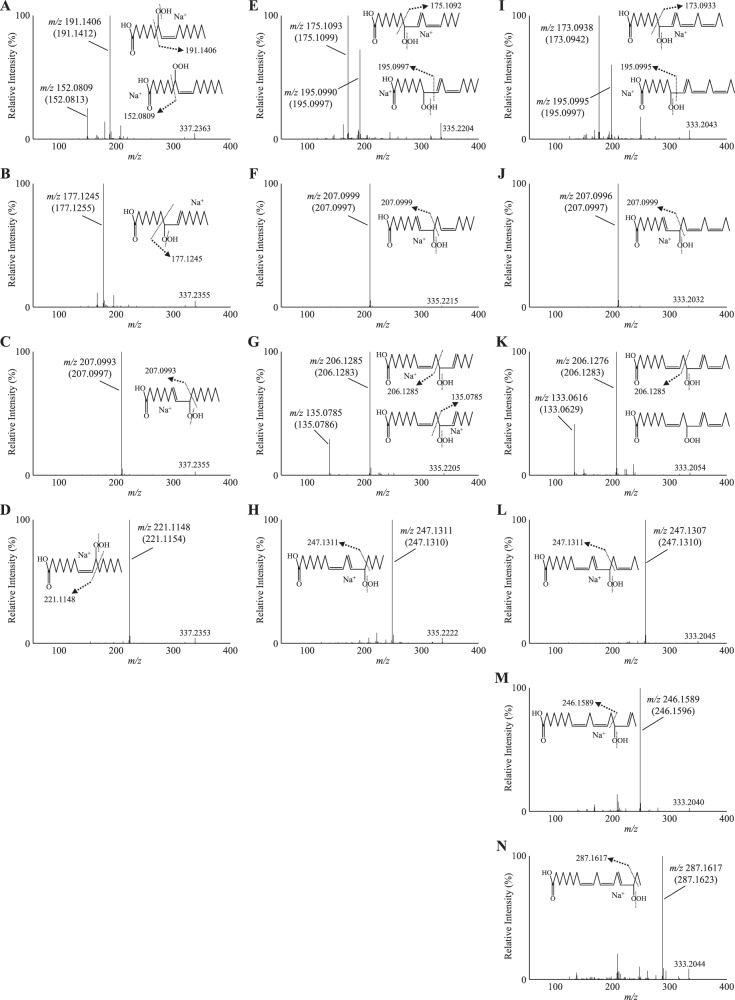
Product ion mass spectra of FAOOH isomers. HpOME isomers (*m/z* 337, **A** 8-HpOME; **B** 9-HpOME; **C** 10-HpOME; and **D** 11-HpOME), HpODE isomers (*m/z* 335, **E** 9-HpODE; **F** 10-HpODE; **G** 12-HpODE and **H** 13-HpODE), and HpOTE isomers (*m/z* 333, **I** 9-HpOTE; **J** 10-HpOTE; **K** 12-HpOTE; **L** 13-HpOTE; **M** 15-HpOTE; and **N** 16-HpOTE).

When the prepared standards were each analyzed by Q1 mass scan, a clear peak was detected at *m/z* 337.2355-337.2379 ([M + Na]^+^) for HpOME, *m/z* 335.2203-335.2232 ([M + Na]^+^) for HpODE and *m/z* 333.2042-333.2051 ([M + Na]^+^) for HpOTE (Supplementary Fig. 2). These *m/z* values were consistent with their theoretical exact mass (*m/z* 337.2355 for HpOME, *m/z* 335.2198 for HpODE and *m/z* 333.2042 for HpOTE). Other ions except for hydroperoxide-related ions (e.g., dehydrated ions and potassium adducts) were not detected, which demonstrated the high purity of the standards. Then, to determine the hydroperoxyl group position of each standard, product ion scan of sodiated FAOOH isomers were conducted. We have previously demonstrated that the collision induced dissociation (CID) of sodiated hydroperoxides (i.e., [M + Na]^+^) provides hydroperoxyl group position specific product ions^21–23^. In accordance with such previous studies, the prepared HpODE isomer standards provided product ions according to the position of the hydroperoxide group (Fig. 2E–H). Additionally, it was newly identified that the CID of HpOME and HpOTE also provided characteristic product ions according to the position of the hydroperoxide group. 8-HpOME ([M + Na]^+^) provided the selective product ions *m/z* 191.1406 and *m/z* 152.0809 (Fig. 2A), and 9-HpOME, 10-HpOME, and 11-HpOME provided product ions *m/z* 177.1245, *m/z* 207.0993, and *m/z* 221.1148, respectively, (Fig. 2B–D). The product ions for HpOTE are described in Fig. 2I–N. The reliability of fragment ions towards the identification of the hydroperoxyl group position was supported by their theoretical exact mass (refer to values in parentheses). Quantification of the prepared standards were performed by the ferrous oxidation-xylenol orange (FOX) assay.

### Investigation of the acrolein generation pathway

Radical oxidation has been assumed to be the primary mechanism by which lipids in edible oils oxidize during thermal treatment. On the other hand, several studies have also suggested that the quality of edible oils deteriorate by daily levels of photo irradiation^24,25^, suggesting the presence of an uncharacterized photo-oxidation pathway. This is supported by our data showing that certain edible oils are susceptible to type II photo oxidation (i.e., ^1^O_2_ oxidation) despite being sealed^18^. However, the mechanisms of ^1^O_2_ oxidation and degradation into aldehydes have not been elucidated.

The FAOOH isomers demonstrated in Fig. 1 represent the isomers that are presumably contained in edible oils that we consume daily. In this study, to investigate whether the decomposition of such FAOOH isomers produces acrolein, the prepared FAOOH standards were individually decomposed at 180 °C. Heating for 30 seconds completely decomposed each FAOOH standard (data not shown). Volatile compounds that emerged during this decomposition were collected with SPME and analyzed by GC-EI-MS. Detected compounds were identified by spectral library comparison and in reference to analysis of a standard (for acrolein, data not shown). Detected volatile compounds included several aldehydes, alcohols, ketones, furans, and hydrocarbons (Table 1).

**Table 1:** Volatile compounds derived from FAOOH isomers.

Peak No.	Compound	Retention time (min)			Peak No.	Compound	Retention time (min)	
1	Propanal	5.8			22	2-Heptenal	28.5	
2	Octane	6.2			23	1-Hydroxy-2-butanone	29.9	
3	1-Octene	7.4			24	Nonanal	30.5	
4	2-Propenal (acrolein)	7.5			25	2-Octenal	31.6	
5	Pentanal	14.4			26	1-Octen-3-ol	32.0	
6	1-Penten-3-one	16.6			27	1-Heptanol	32.1	
7	2-Butenal	17.5	17.8		28	2,4-Heptadienal	32.5	33.3
8	Hexanal	19.5			29	Formic acid	33.1	
9	1,3,5-Octatriene	20.3			30	2-Nonenal	34.5	
10	2-Pentenal	20.5	21.4		31	1-Ocatanol	34.9	
11	3-Penten-2-one	21.3			32	5-Ethyl-2-furanone	35.8	
12	3-Hexenal	22.0			33	2-Octen-1-ol	36.2	36.3
13	1-Penten-3-ol	22.8			34	2-Decenal	37.3	
14	Heptanal	23.7			35	*n*-Caproic acid vinyl ester	37.7	
15	2,4,6-Octatriene	23.7			36	2-Undecenal	39.9	
16	2-Hexenal	24.7			37	4-Oxohex-2-enal	40.1	
17	2-Pentylfuran	25.3			38	2,4-Decadienal	40.6	41.2
18	1-Pentanol	26.0			39	Hexanoic acid	41.6	
19	Octanal	27.3			40	Heptanoic acid	43.9	
20	1-Octen-3-one	27.6			41	4,5-Epoxy-2-decenal	44.9	45.2
21	2-Penten-1-ol	27.9	28.2		42	Octanoic acid	46.1	

**Fig. 3:**
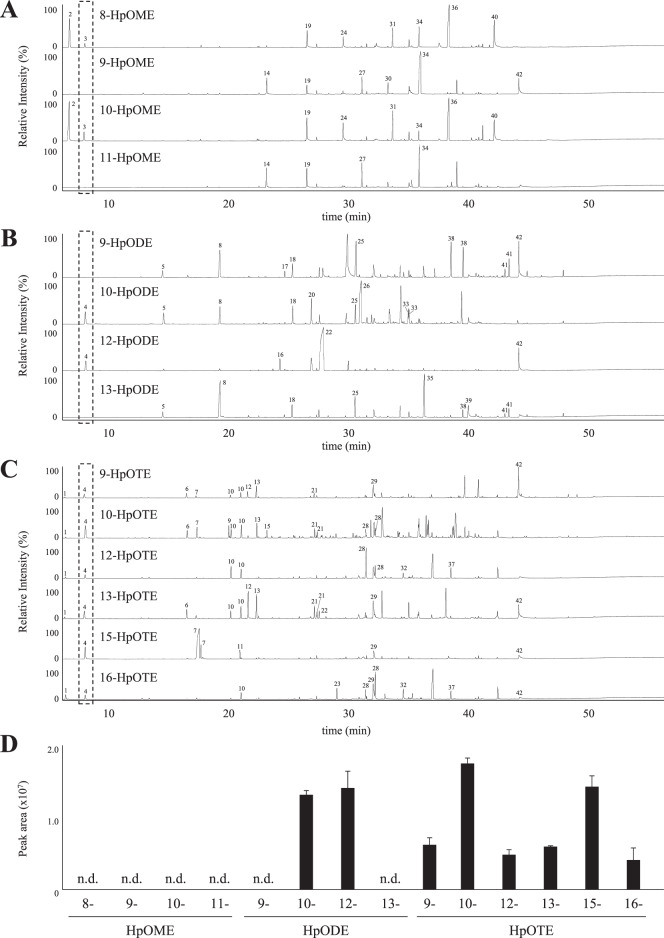
Volatile compounds derived from each FAOOH isomer. GC-EI-MS chromatograms of volatile compounds (**A** HpOME; **B** HpODE; and **C** HpOTE). Identified compounds (peak number 1–42) are summarized in Table 1. The amount of acrolein generated from each FAOOH isomer (**D**). Data are expressed as means ± SDs (*n* = 3).

The decomposition of HpOME isomers did not generate acrolein (Fig. 3A and Table 1). This was in agreement with a previous study which demonstrated that the heating of trioleate formed only trace levels of acrolein^17^. Acrolein was also not generated by the decomposition of 9- and 13-HpODE, the main HpODE isomers formed by radical oxidation (Fig. 3B and Table 1). This was also in agreement with a previous study which demonstrated that heating of trilinoleate formed only trace levels of acrolein^17^. On the other hand, decomposition of ^1^O_2_ oxidation-specific HpODE isomers (i.e., 10- and 12-HpODE) generated a significant amount of acrolein (Fig. 3B and Table 1). To the best of our knowledge, this is the first study suggesting that LA can be a source of acrolein under photo irradiation.

The decomposition of HpOTE isomers each resulted in the formation of acrolein (Fig. 3C and Table 1). Notably, the decomposition of ^1^O_2_ oxidation-specific HpOTE isomers (i.e., 10- and 15-HpOTE) generated a 2–3-fold higher amount of acrolein compared with the decomposition of other HpOTE isomers (Fig. 3D). These results clearly demonstrate that the amount of acrolein generated as well as the pathway leading to its formation depends on the FA species and hydroperoxyl group position of FAOOH. Moreover, the results suggested that the decomposition of ^1^O_2_ oxidation-specific FAOOH isomers produce larger amounts of acrolein compared with other FAOOH isomers.

**Fig. 4:**
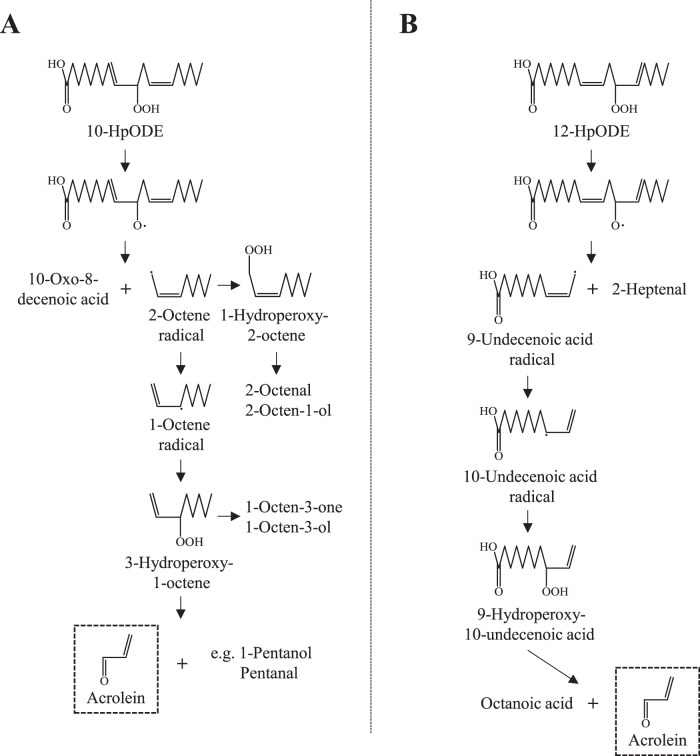
Proposed pathways leading to the generation of acrolein. 10-HpODE (**A**) and 12-HpODE (**B**).

Based on the above results, the pathway leading to the formation of acrolein was evaluated. It is well-known that the first reaction of the hydroperoxide decomposition process is the formation of alkoxyl radicals caused by the weakness of the O–OH bond^20^. One of the next reactions is a β-scission reaction by the alkoxyl radical^20^. As to which side is cleaved (i.e., methyl- or carbonyl-side C–C), β-scission prefers the reaction, which generates compounds that possess a more stable structure (e.g., resonance or conjugated structure)^26,27^. For example, β-scission of 10-HpODE prefers the cleavage between C10 and C11, which affords 10-oxo-8-decenoic acid, a stable conjugated aldehyde, and 2-octene radical, an aryl radical (Fig. 4A). The resultant 2-octene radical is further oxidized to 1-hydroperoxy-2-octene, followed by reduction to either 2-octenal or 2-octen-1-ol. The 2-octene radical can also be rearranged to a 1-octene radical via the delocalization of the radical, and can be further oxidized to 3-hydroperoxy-1-octene. 3-Hydroperoxy-1-octene can either be reduced to 1-octene-3-one and 1-octene-3-ol or undergo a stable β-scission reaction to afford acrolein. Of the above reactions, generation of a 3-hydroperoxy-1-alkene, which occurs via a β-scission, radical delocalization, and a further peroxidation, is the most important pathway for the formation of acrolein. And, detection of some byproducts supported this pathway; 2-octenal, 2-octen-1-ol, 1-octen-3-one (1-alken-3-one), 1-octen-3-ol (1-alken-3-ol), 1-pentanol, and pentanal were detected by GC-MS when the decomposition products of 10-HpODE were analyzed (Fig. 3B and Table 1). A previous study reporting that 1-alken-3-one (i.e., 1-penten-3-one) and 1-alken-3-ol (1-penten-3-ol) are generated together with acrolein by thermal oxidation of 7,10,13,16,19-docosapentaenoic acid ethyl ester^28^ supports the above pathway. Similarly with 10-HpODE, the decomposition of 12-HpODE initially produces a conjugated aldehyde (2-heptenal) and an aryl radical (9-undecenoic acid radical) via a β-scission reaction between C11 and C12 (Fig. 4B and Table 1). The preferential cleavage between C11 and C12 was supported by the detection of a significant amount of 2-heptenal (Fig. 3B and Table 1). The 9-undecenoic acid radical undergoes a rearrangement to afford a 10-undecenoic acid radical, followed by the peroxidation to 9-hydroperoxy-10-undecenoic acid (corresponding to a 3-hydroperoxy-1-alkene). 9-Hydroperoxy-10-undecenoic acid then breaks down to acrolein via a preferential β-scission reaction between C8 and C9. In addition to acrolein, this cleavage generates octanoic acid, which was detected on the GC-MS chromatogram (Table 1). The above results demonstrate that the acrolein generation pathway can be explained by the hydroperoxyl group position, β-scission position, and radical delocalization. In light of these factors, it can be perceived that the decomposition of compounds with the partial structure of –CH=CH–CH(OOH)–CH_2_–CH=CH– can generate acrolein via a β-scission reaction, peroxidation (generation of 3-hydroperoxy-1-alkene) followed by another β-scission reaction. Notably, this structure is formed in larger amounts by ^1^O_2_ oxidation than radical oxidation in all polyunsaturated FA (PUFA; e.g., LnA, arachidonic acid, and docosahexaenoic acid). Hence, the pathway suggests that the photo irradiation of PUFA increases the amount of acrolein produced.

**Fig. 5:**
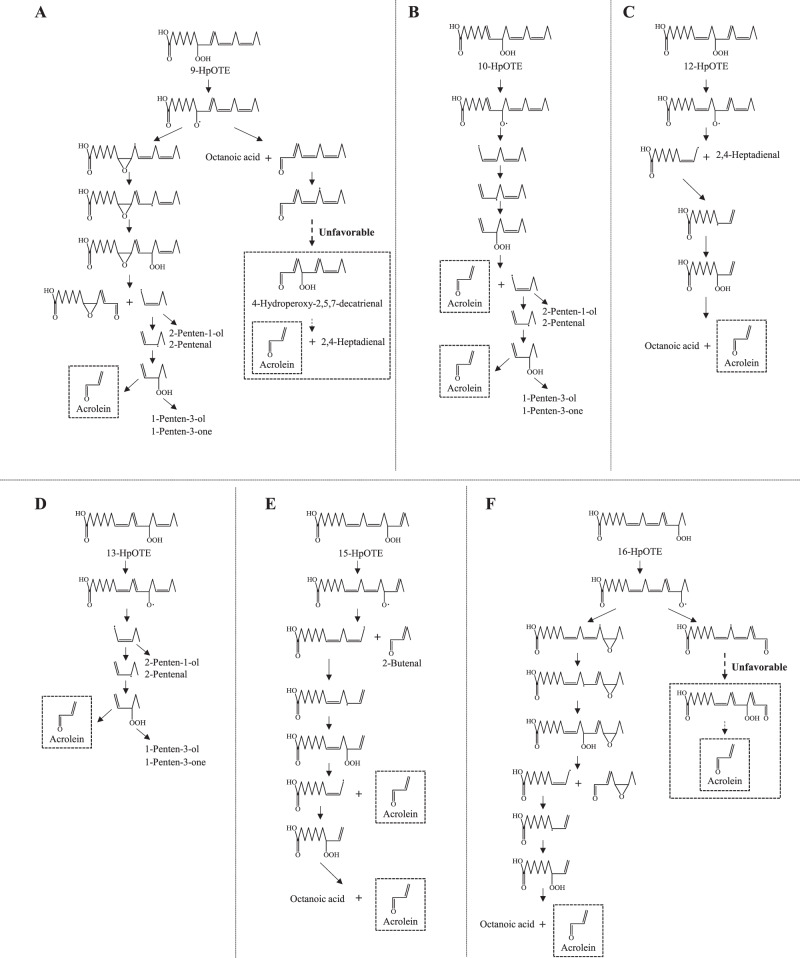
Proposed pathways leading to the generation of acrolein. 9-HpOTE (**A**), 10-HpOTE (**B**), 12-HpOTE (**C**), 13-HpOTE (**D**), 15-HpOTE (**E**), and 16-HpOTE (**F**).

Taking into account the factors and pathways identified above, the acrolein formation pathway from HpOTE isomers was also evaluated. As shown in Fig. 5, it was estimated that the HpOTE isomers generated by radical oxidation (i.e., 9-, 12-, 13-, and 16-HpOTE) each potentially generate one molecule of acrolein per a HpOTE molecule. The decomposition pathways of 12- and 13-HpOTE were considered to be similar to those of 12- and 10-HpODE, respectively.

Additionally, epoxidation was considered to be required for acrolein formation from 9- and 16-HpOTE (Fig. 5A, F). As described in previous studies^19,20,26,27^, alkoxy radical adds to α-carbon to form an epoxides along with transfer of the free electron to the β-carbon. For instance, the alkoxy radical of 9-HpOTE affords a 9,10-epoxy allylic radical, followed by oxidation to 9,10-epoxy-13-hydroperoxide (Fig. 5A). A β-scission reaction of 9,10-epoxy-13-hydroperoxide affords acrolein via the formation of a 3-hydroperoxy-1-alkene. Detection of byproducts (2-penten-1-ol, 2-pentenal, 1-penten-3-ol, and 1-penten-3-one) supported this pathway. With regard to the formation of acrolein from LnA, several studies have proposed the pathway by which acrolein is generated from the β-scission of 4-hydroperoxy-2-alkenal (e.g., 4-hydroperoxy-2,5,7-decatrienal (Fig. 5A))^12–17^. However, the generation of 4-hydroperoxy-2-alkenal is likely unfavorable, since insertion of a hydroperoxyl group at C4 of 2,4,7-decatrienal requires decomposition of a stable resonance structure, as supported by the trace detection of 2,4-heptadienal from 9-HpOTE in this study (Fig. 3C and Table 1). During the decomposition of ^1^O_2_ oxidation-specific HpOTE isomers (i.e., 10- and 15-HpOTE), two molecules of acrolein per a HpOTE molecule should be formed because these isomers afford the –CH = CH–CH(OOH)–CH_2_–CH = CH– structure twice during the decomposition pathway. This estimation was supported by the detection of 2–3-fold higher amounts of acrolein from the decomposition of 10- and 15-HpOTE compared with other HpOTE isomers (Fig. 3D).

Meanwhile, the predicted pathways estimated that the amount of acrolein derived from 10-HpODE, 12-HpODE, 9-HpOTE, 12-HpOTE, 13-HpOTE, and 16-HpOTE should be one molecule per a FAOOH molecule, and that the amount of acrolein derived these isomers should be two times lower than that of 10- and 15-HpOTE. However, the amount of acrolein derived from 10- and 12-HpODE was two times higher than that of 9-, 12-, 13-, and 16-HpOTE and was equivalent to that of 10- and 15-HpOTE. This reduction in the formation of acrolein from HpOTE isomers may be due to the following reasons: the 1,4-pentadiene structure of HpOTE (Fig. 5A, B, E and F) may participate in other reactions (e.g., generation of a stable conjugated di(tri)ene) via further abstraction of a proton radical and/or an intramolecular reaction between alkoxyl radicals and the γ-carbon may form furans (Fig. 5A–F).

Overall, an important discovery derived from the pathway was the fact that ^1^O_2_ oxidation-specific HpODE isomers could be a source of acrolein. Furthermore, decomposition of ^1^O_2_ oxidation-specific HpOTE isomers generated a higher amount of acrolein compared with that of other HpOTE isomers. Hence, a significant contribution of photo irradiation towards the generation of acrolein from edible oils is proposed.

The above results demonstrate a novel pathway whereby acrolein is generated via the decomposition of FAOOH. While the described pathway is only one of the possible reactions that takes place among other reactions (e.g., Hock fragmentation^29^), the detection of intermediate and byproducts suggests the relevance of this photo-oxidation pathway. Some peaks could not be identified despite their significant intensity (especially on chromatograms of HpOTE decomposition products, Fig. 3), indicating that the characterization of these compounds in future studies could add more confirmatory data to the proposed pathway.

### Analysis of acrolein derived from photo-irradiated edible oils

The above results strongly suggested that the presence of ^1^O_2_ oxidation-specific HpODE (i.e., 10- and 12-HpODE) and HpOTE (i.e., 10- and 15-HpOTE) isomers increase the amount of acrolein formed. Considering our previous report demonstrating that the irradiation of edible oils with daily levels of light induce ^1^O_2_ oxidation^18^, the use of such edible oils oxidized by ^1^O_2_ oxidation would increase the exposure and dietary intake of acrolein. Therefore, we then investigated whether photo irradiation increases the amount of acrolein generated from edible oils. Since this study newly suggested that LA is a potential source of acrolein, edible oils that possess different compositions of LA were used: rapeseed oil (OA: 65.2%, LA: 19.5%, LnA: 7.7%), rice bran oil (OA: 44.9%, LA: 34.7%, LnA: 1.0%), and soybean oil (OA: 23.2%, LA: 54.9%, LnA: 6.6%)^10^. Edible oils were each oxidized by pre-irradiation under 5000 lux (16–18 °C) for 0–7 days assuming the storage of edible oils on shelves on the market or at homes. Subsequently, the oxidized oils were heated at 180 °C for 90 seconds.

**Fig. 6:**
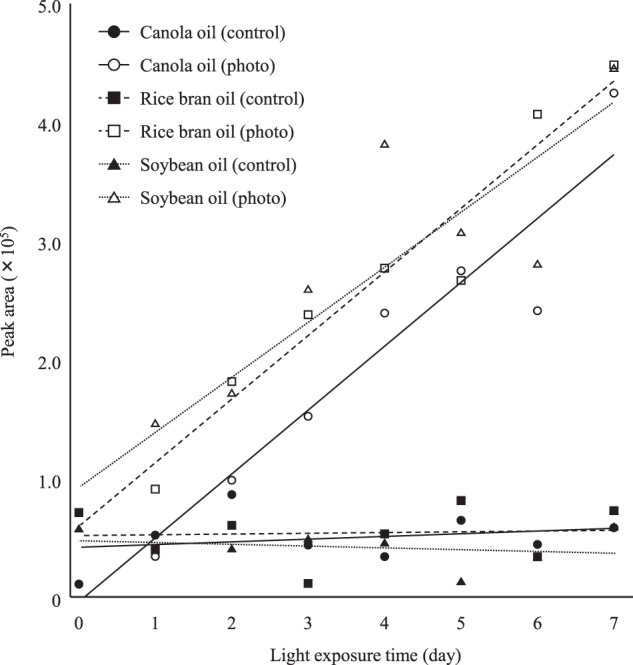
Acceleration of acrolein generation in edible oils by photo irradiation. Photo-irradiated (5000 lux for 0–7 days) edible oils were heated.

Edible oils placed in the dark did not produce acrolein by the heating process (Fig. 6). On the other hand, as we expected, the amount of acrolein generated from photo-irradiated edible oils increased with pre-irradiation time. Importantly, rice bran oil generated acrolein in a comparable amount to other oils despite its low LnA content, suggesting that the source of acrolein was mainly ^1^O_2_ oxidation-specific HpODE isomers (i.e., 10- and 12-HpODE). On the other hand, the amount of acrolein derived from soybean oil was similar to that of other edible oils despite its high LA and LnA content. This may be attributed to the relatively high content of photosensitizers (e.g., chlorophyll) that initiate the photo-oxidation (^1^O_2_ oxidation) process^30^. Accordingly, in addition to FA composition, the content of photosensitizers may also affect the generation of acrolein from edible oils.

Storage and culinary processes during the daily use of edible oils expose them to light. Therefore, antioxidative measures to prevent photo oxidation (^1^O_2_ oxidation), for example, the addition of ^1^O_2_ trapping reagents (e.g., carotenoids) and improvement of packaging techniques (e.g., use of amber bottles), may contribute to the reduction of acrolein formed from edible oils and to dietary exposure.

In summary, in this study, we sought to determine the mechanism of acrolein generation from ^1^O_2_ and radical oxidation. Standards of the main FAOOH isomers (HpOME, HpODE, and HpOTE) contained in edible oils were prepared, and their thermal decomposition products were analyzed. Our results show that the decomposition of ^1^O_2_ oxidation-specific HpODE isomers (i.e., 10- and 12-HpODE) generated a significant amount of acrolein. To the best of our knowledge, this is the first study reporting that LA can be a significant source of acrolein. Consistent with this observation, edible oils rich in LA (e.g., rice bran oil) increased acrolein formation with pre-irradiation despite its low LnA content. Furthermore, from the analysis of other volatile compounds, we found that the acrolein generation pathway can be explained by FA species, oxidation mechanisms (i.e., hydroperoxyl group position), β-scission position, and radical delocalization. Based on this pathway, it was estimated that the amount of acrolein derived from ^1^O_2_ oxidation-specific HpOTE isomers (i.e., 10- and 15-HpOTE) was two times greater than that of other HpOTE isomers. Hence, the results of this study provide valuable insights into the improvement of food storage and processing strategies that would ultimately contribute to product shelf-life stability and reduced dietary exposure to acrolein.

## Materials and methods

### Materials

OA and LnA were obtained from Sigma Aldrich, Inc (Darmstadt, Germany) and Nu-Chek Prep, Inc. (Elysian, MN, USA), respectively. LA, MeO-AMVN, and rose bengal were from FUJIFILM Wako Pure Chemical Corp. (Osaka, Japan). Acrolein was obtained from AccuStandard (New Haven, CT, USA). Marketed edible oil products (rapeseed oil, rice bran oil, and soybean oil) were purchased at a local market in Sendai, Japan. All other reagents were of the highest grade available.

### Preparation of HpOME, HpODE, and HpOTE isomer standards

OA (15 g) was mixed with 185 mg of MeO-AMVN. The mixture was placed at 45 °C for 6 h. After the reaction, the crude oxidized OA was dissolved in methanol. To remove secondary oxidation products, this crude radical oxidation product of OA was injected to a reverse phase semipreparative HPLC system (Shimadzu, Kyoto, Japan). An UV/Vis detector was set at 210 nm. HpOME fraction was isolated using an ODS column (Inertsil ODS-3, 10 µm, 20 × 250 mm, GL Sciences Inc., Tokyo, Japan) with methanol/water/acetic acid (100:20:0.12, v/v/v) as the mobile phase. The flow rate was set at 20 mL/min and the column was maintained at 40 °C. The HpOME fraction was further purified to each HpOME isomer by normal phase HPLC. HpOME isomers were separated using an Inertsil SIL-100A column (5 μm, 10 × 250 mm, GL Sciences Inc., Tokyo, Japan) with hexane/2-propanol/acetic acid (100:1:0.1, v/v/v) as the mobile phase. The flow rate was set at 10 mL/min and the column was maintained at 40 °C. Purification by normal phase HPLC was repeated several times to refine the purity of each isomer. The purified standards were dissolved in chloroform.

LA (2 g) and LnA (2 g) were oxidized by photo oxidation. Each FA was dissolved in 50 mL of methanol to which rose bengal (500 µg) was added. Each solution was exposed to light-emitting diode irradiation (LED, 50,000 lux, 4 °C) for 15 h. After oxidation, rose bengal was removed using a Sep-Pak Vac QMA column (3 cc, 500 mg, Waters, MA, USA), and the solutions were evaporated under a nitrogen gas stream. The samples were dissolved in hexane and injected to a normal phase semipreparative HPLC system to obtain HpODE isomers and HpOTE isomers under the same conditions as described above.

The concentration of purified FAOOH isomers were analyzed by the FOX assay as described previously^31^. The standards were stored at −80 °C until use.

### Determination of hydroperoxyl group positions by time of flight (TOF)-MS

Each FAOOH standard was dissolved in methanol (10 μM). To determine the hydroperoxyl group position of each FAOOH isomer, each solution was directly injected to a TOF-MS system (micrOTOF-Q II, Bruker Daltonics, GmbH, Bremen, Germany) at 5 μL/min. The TOF-MS system was operated using the micrOTOF Control Software (ver. 3.2) (Supplementary Table 1). Mass spectra were obtained in the scan range of *m/z* 50–400.

### Thermal decomposition of FAOOH isomers

FAOOH standards (50 nmol) were individually placed into an amber glass vial (2 mL Sc-Vial, GL science, Tokyo, Japan) (*n* = 3). The solvent was thoroughly evaporated under reduced pressure. Each vial was then sealed with screw cap under ambient air. Decomposition of FAOOH was performed by placing the vial on a heating plate (EC-1200N, AS ONE, Osaka, Japan) set at 180 °C for 30 seconds. After heating, the vial was immediately cooled on ice. Headspace gas, which contained volatile compounds, was analyzed by GC-EI-MS as described below.

### GC-EI-MS analysis

Volatile compounds were collected with SPME. A StableFlex 50/30 µm Divinylbenzene/Carboxen/Polydimethylsiloxane fiber (SUPELCO, Bellefonte, PA, USA) was exposed to the vial headspace for 20 min at 40 °C. After collection, analytes were desorbed into a GC-EI-MS system (GCMS-QP2010 SE, Shimadzu, Kyoto, Japan). Desorption was conducted in the splitless mode at 250 °C for 4.5 min. Volatile compounds were separated on a DB-WAX-UI column (60 m, 0.25 mm i.d., 0.25 μm film thickness; Agilent Technologies, CA, USA). For the analysis of standard decomposition, the initial temperature was set at 30 °C, held for 10 min, ramped to 250 °C at 5 °C/min and held for 5 min. In the analysis of volatile compounds from edible oils, the initial temperature was set at 30 °C, held for 10 min, ramped to 250 °C at 50 °C/min and held for 15 min. Helium was used as the carrier gas with a constant linear velocity of 25 cm/sec. EI-MS spectra were obtained in the range of *m/z* 41–500 at the ion source temperature of 250 °C. Compounds were identified by spectral comparison to the NIST 17 mass spectral library using the GCMS solution software (ver. 4.50).

### Analysis of marketed edible oils

Marketed edible oils (rapeseed oil, rice bran oil, and soybean oil, 1 g) were placed in transparent vials (FIOLAX, klar HGB 1/ISO (719 52.00 × 22,00/1.20 mm), NIPRO, Dedecke GmbH, Königswinter, Germany) (*n* = 1). The vial was sealed under ambient air and oxidized under LED irradiation (5000 lux) at 16–18 °C for 0–7 days. After irradiation, each vial was heated at 180 °C for 90 seconds. After heating, the vials were immediately cooled on ice. Volatile compounds were collected and analyzed by GC-EI-MS as described above.

## Supplementary information

Preparation of fatty acid hydroperoxide isomers

## Data Availability

Raw data were generated at Tohoku University. Derived data supporting the findings of this study are available from the corresponding author on request.
