# Supplementary material for: Determination of acrolein generation pathways from linoleic acid and linolenic acid: increment by photo irradiation
Source: NPJ Sci Food. 2022 Apr 12;6:21. doi: 10.1038/s41538-022-00138-2 (PMC9005701; doi:10.1038/s41538-022-00138-2)
Supplement: Supplementary file 1 — Preparation of fatty acid hydroperoxide isomers [file 41538_2022_138_MOESM1_ESM.pdf]

Supplementary Information 1

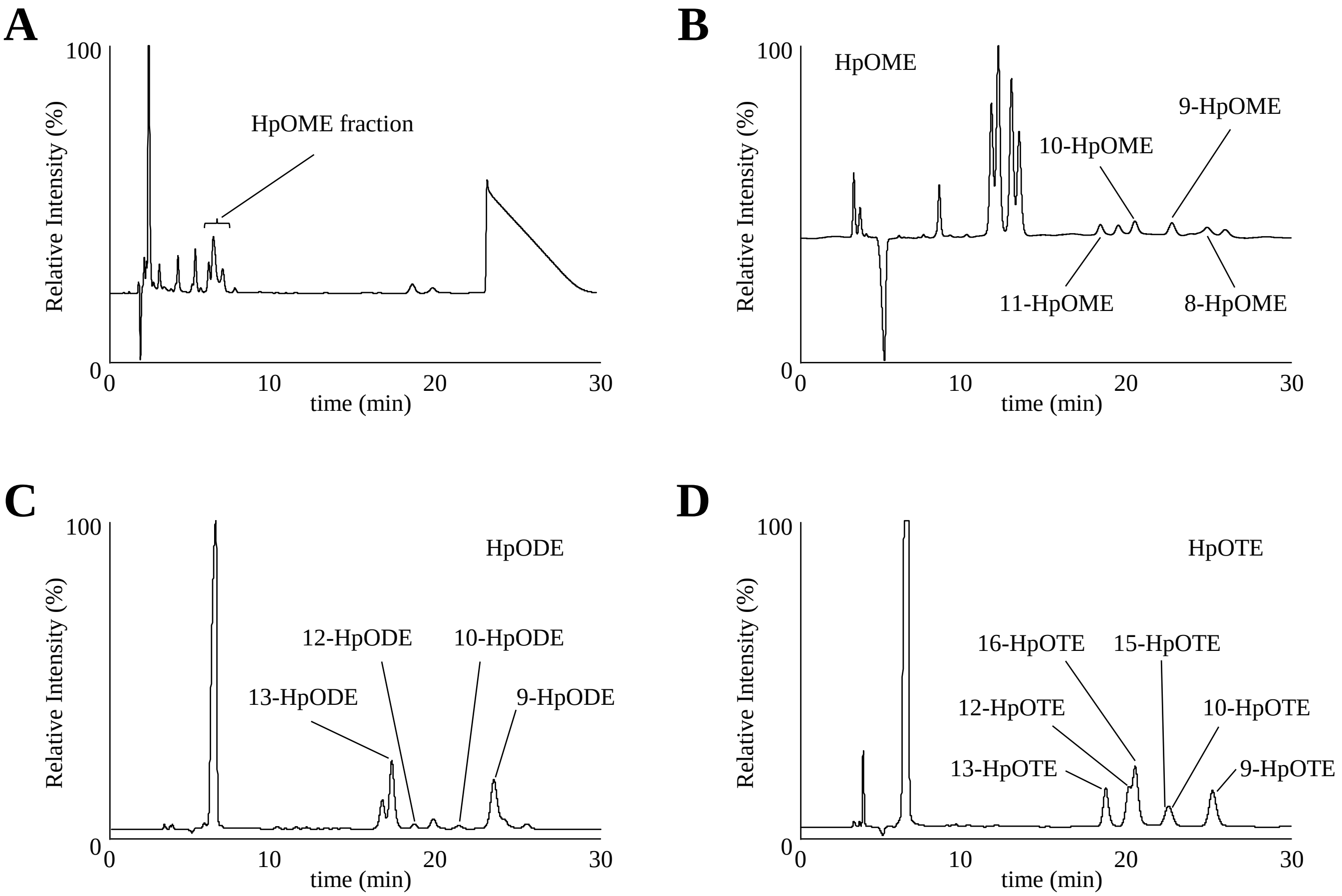

UV chromatograms for the preparation of FAOOH isomers. For the HpOME preparation, a crude radical oxidation product of OA was firstly purified with reverse phase-HPLC (**A**). Obtained HpOME fraction was further purified to each HpOME isomer by normal phase-HPLC (**B**). $^1O_2$ oxidized LA and LnA were directly purified by normal phase HPLC to HpODE (**C**) and HpOTE (**D**) isomers, respectively.

Supplementary Information 2

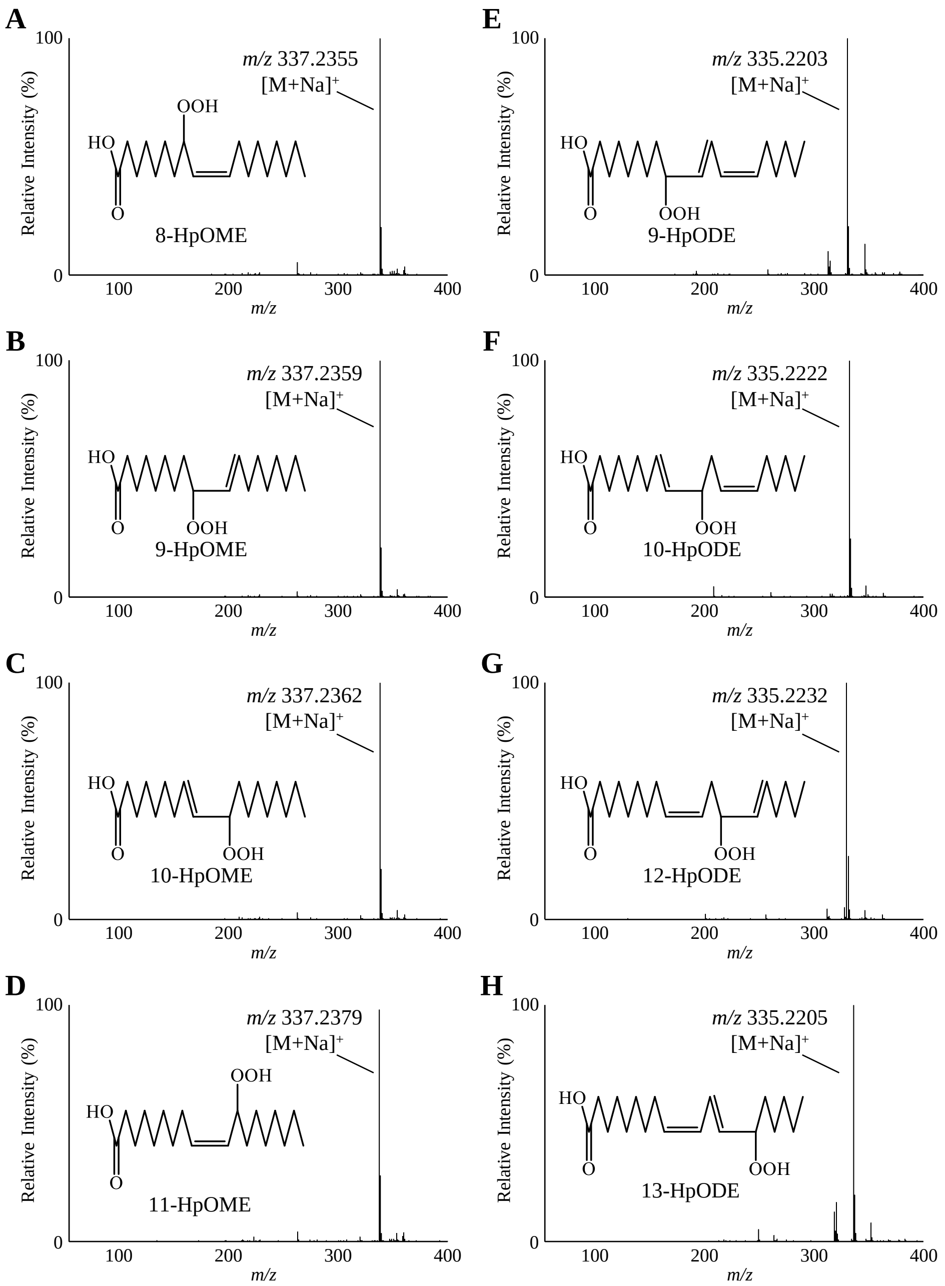

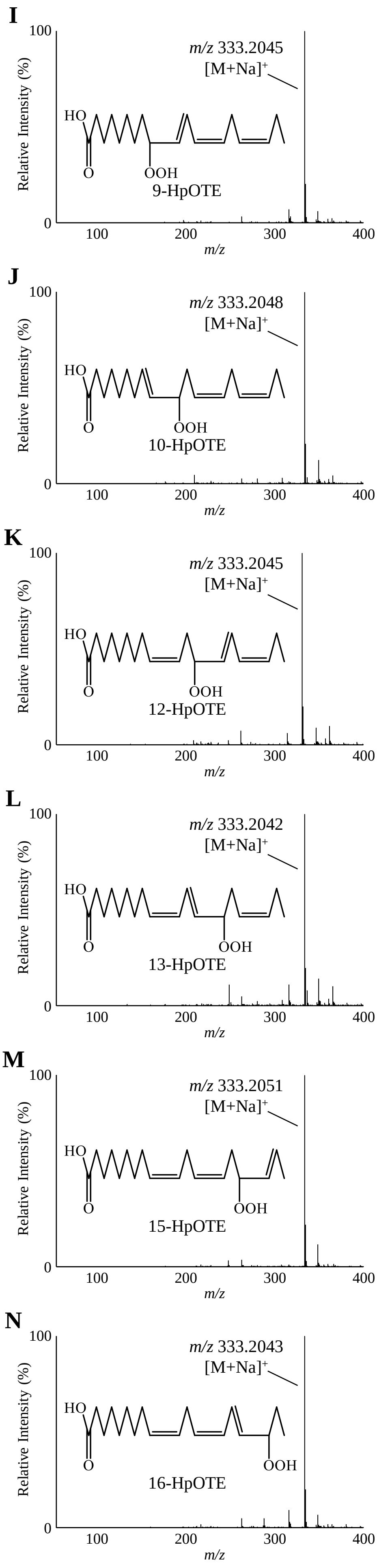

Q1 mass spectra of HpOME isomers (*m/z* 337, **A**, 8-HpOME; **B**, 9-HpOME; **C**, 10-HpOME and **D**, 11-HpOME), HpODE isomers (*m/z* 335, **E**, 9-HpODE; **F**, 10-HpODE; **G**, 12-HpODE and **H**, 13-HpODE) and HpOTE isomers (*m/z* 333, **I**, 9-HpOTE; **J**, 10-HpOTE; **K**, 12-HpOTE; **L**, 13-HpOTE; **M**, 15-HpOTE and **N**, 16-HpOTE).

Supplementary Information 3

Supplementary Information 3 MS/MS parameters for the detection of product ions

	HpOME	HpODE	HpOTE
Precursor ion (*m/z*)	337.24	335.22	333.20
End plate offset (V)	500	500	500
Capillary (V)	5000	5000	5000
Nebulizer (Bar)	0.4	0.4	0.4
Dry Gas (L/min)	4.0	4.0	4.0
Dry Temp (°C)	180	180	180
Funnel 1 RF (Vpp)	100	100	100
Funnel 2 RF (Vpp)	100	100	100
Hexapole RF (Vpp)	200	200	200
Ion Energy (eV)	3.0	3.0	3.0
Collision RF (Vpp)	200	200	200
Transfer Time (µs)	35	35	35
Pre pulse storage (µs)	8.0	8.0	8.0
isCID energy (eV)	0	0	0
Collision energy (eV)	16	16	16
